# Conformal Prediction Based on K-Nearest Neighbors for Discrimination of Ginsengs by a Home-Made Electronic Nose

**DOI:** 10.3390/s17081869

**Published:** 2017-08-14

**Authors:** Zhan Wang, Xiyang Sun, Jiacheng Miao, You Wang, Zhiyuan Luo, Guang Li

**Affiliations:** 1 State Key Laboratory of Industrial Control Technology, Institute of Cyber Systems and Control, Zhejiang University, Hangzhou 310027, China; 11732003@zju.edu.cn (Z.W.); 21532085@zju.edu.cn (X.S.); jiacheng@zju.edu.cn (J.M.); guangli@zju.edu.cn (G.L.); 2 Computer Learning Research Centre, Royal Holloway, University of London, Egham Hill, Egham, Surrey TW20 0EX, UK; zhiyuan@cs.rhul.ac.uk

**Keywords:** conformal prediction, electronic nose, k-nearest neighbors, ginseng

## Abstract

An estimate on the reliability of prediction in the applications of electronic nose is essential, which has not been paid enough attention. An algorithm framework called conformal prediction is introduced in this work for discriminating different kinds of ginsengs with a home-made electronic nose instrument. Nonconformity measure based on k-nearest neighbors (KNN) is implemented separately as underlying algorithm of conformal prediction. In offline mode, the conformal predictor achieves a classification rate of 84.44% based on 1NN and 80.63% based on 3NN, which is better than that of simple KNN. In addition, it provides an estimate of reliability for each prediction. In online mode, the validity of predictions is guaranteed, which means that the error rate of region predictions never exceeds the significance level set by a user. The potential of this framework for detecting borderline examples and outliers in the application of E-nose is also investigated. The result shows that conformal prediction is a promising framework for the application of electronic nose to make predictions with reliability and validity.

## 1. Introduction

Two main problems that the electronic nose (E-nose) faces are classification and regression. Lots of techniques, such as support vector machine (SVM) [1], k-nearest neighbors (KNN) [2,3,4], artificial neural network (ANN) [5,6], linear discriminant analysis (LDA) [2,3,4,7,8] and other methodologies, have been successfully applied for predictions with E-nose. However, there are still two drawbacks: (1) the lack of reliable measure of the confidence in their individual prediction; and (2) the accuracy of overall prediction is not guaranteed. Many approaches have been developed to complement these drawbacks by predicting with additional information, such as probably approximately correct learning (PAC), Bayesian learning, generalized least square regression in combination with a stepwise backward selection [9] and hold-out estimate or cross-validation.

In PAC learning, upper bounds on the probability of error often exceed one even for a relatively clean data set, which makes it useless in practical applications. In addition, PAC learning does not provide any information on the reliability of individual prediction [10]. In contrast, Bayesian learning and other probability algorithms, such as logistic regression [11] and Platt’s method [12], can complement every individual prediction with information of probability to indicate how every potential label is correct. However, the main disadvantage of these algorithms is that they depend on a strong statistical assumption for the model. Once the data does not conform well to statistical model, the prediction may be invalid and misleading. Data generated from E-nose is usually difficult to be described by a precise statistical model because most gas sensors suffer from sensor drift caused by variation of temperature and humidity, sensor aging, lack or partial selectivity and sensor poisoning [13,14]. Though lots of work have been devoted to drift calibration, there is still no effective approach to compensate the drift [15,16,17]. Hold-out estimate and cross-validation provide information about the accuracy of overall prediction. Usually, the dataset is divided into training set and testing set; then, the average prediction accuracy of the testing set is used to estimate the performance of the model. However, this performance estimation usually provides overoptimistic results in real-world applications, since testing samples measured afterwards may suffer from sensor drift, sensor aging or sensor poisoning. ‘External validation’ is strongly recommended for artificial olfaction by some researchers, where other testing sets generated in different experiment conditions from the training set should be used [18]. However, the performance of ‘external validation’ is still unsure and also depends on the experiment conditions. Therefore, the accuracy of overall prediction cannot be guaranteed by hold-out estimate or cross-validation.

Conformal prediction was proposed and developed by Vladimir Vovk and his co-workers since 2005 [10,11,19,20]. It is based on a consistent and well-defined mathematical framework and measures how well the new example is conformed to the group of observations. The algorithm produces each individual prediction with additional information of confidence and credibility. The most important property of conformal prediction is automatic validity under the randomness assumption: the objects and their labels are assumed to be generated from the identical probability distribution. Informally, validity means that conformal predictor never overrates the accuracy and reliability of their prediction. The randomness assumption is a much weaker condition than statistical assumption and can easily be satisfied by real-world data, such as E-nose data.

In this paper, a home-made electronic nose is introduced to discriminate nine different kinds of ginsengs and conformal prediction is used to make reliable and valid predictions. The definition of conformal prediction and nonconformity measure is introduced in Section 2. Then, the sampling experiment with a homemade E-nose and data pre-processing process is presented in Section 3. The results and discussions are shown in Section 4. Finally, we draw the conclusions of our research in Section 5.

## 2. Conformal Prediction

### 2.1. Definition

Let **X** be a measurable space (the object space) and **Y** be a finite set (the label space). Every sample, $z_{i}=\left(x_{i},y_{i}\right)$, is composed of a object $x_{i}\in X$ and a label $y_{i}\in Y$. The observation space is defined as Z:=X∗Y, and $z_{i}\in Z$. We find a measurable function A that changes every sequence of observations, $(z_{1},z_{2},\ldots ,z_{n})\in Z$, to a same-length sequence $(\alpha_{1},\ldots \alpha_{n})\in R$, which is formed by positive real numbers and is equivariant with respect to permutations: for any *n* and any permutation π of {1,…,n}

$$\left(\alpha_{1},\ldots ,\alpha_{n}\right)=A\left(z_{1},\ldots ,z_{n}\right)\to \left(\alpha_{\pi (1)},\ldots ,\alpha_{\pi (n)}\right)=A\left(z_{\pi (1)},\ldots ,z_{\pi (n)}\right).$$

The conformal predictor determined by A meets the exchangeability assumption and is defined by

(1) $$\Gamma^{\epsilon }\left(z_{1},\ldots ,z_{l},x\right):=\left\{y|p^{y}>\epsilon \right\},$$

where $(z_{1},\ldots ,z_{l})\in Z$ is a training sequence that is a part of observation space $\left(z_{1},z_{2},\ldots ,z_{n}\right)$, x is a test object, and y∈Y is potential labels for x. In this work, E-nose was used to deal with classification problem, so Y is the set of labels of all sample categories, $\Gamma^{\epsilon }$ is a corresponding predict region with a given *significance level* ϵ∈(0,1) . For each y∈Y , the corresponding *p*-value is defined by

(2) $$p^{y}=\frac{\left|\{i=1,\ldots ,l+1|\alpha_{i}^{y}\geq \alpha_{l+1}^{y}\}\right|}{l+1}.$$

The corresponding sequence of *nonconformity* scores is defined by

(3) $$\left(\alpha_{1}^{y},\ldots ,\alpha_{l}^{y},\alpha_{l+1}^{y}\right)=A\left(z_{1},\ldots ,z_{l},\left(x,y\right)\right).$$

Generally speaking, the lower the $\alpha_{l+1}^{y}$ is, the more confidence we have. The lower the $p^{y}$ is, the less we can trust this prediction.

It is clearly that the predict region in a conformal predictor is nested, i.e., for any $\epsilon_{1}<\epsilon_{2}$,

(4) $$\Gamma^{\epsilon_{2}}\left(z_{1},\ldots ,z_{l},x\right)\subseteq \Gamma^{\epsilon_{1}}\left(z_{1},\ldots ,z_{l},x\right).$$

The property of validity of conformal predictor is that for any l, the probability of the event

$$y_{l+1}\in \Gamma^{\epsilon }\left(z_{1},\ldots ,z_{l},x_{l+1}\right)$$

is at least 1−ϵ, i.e.,

(5) $$P(y_{l+1}\notin \Gamma^{\epsilon }\left(z_{1},\ldots ,z_{l},x_{l+1}\right))\leq \epsilon .$$

### 2.2. Nonconformity Measure

In theory, any other prediction algorithms can be modified and developed as underlying algorithms (nonconformity measure). Vladimir Vovk once used k-nearest neighbors as the underlying algorithm to compute nonconformity measure for the conformal prediction [10]; so, in this work, we implement a typical nonconformity measure based on KNN. For simplicity, we define CP-1NN and CP-3NN as conformal predictor based on 1NN and 3NN, while simple predictors are 1NN and 3NN.

Given a sequence of examples $\left(z_{1},\ldots ,z_{n}\right)$, the nonconformity score measure by CP-KNN is

(6) $$\alpha_{i}:=\frac{\sum_{j=1}^{k}d_{\mathrm{ij}}^{+}}{\sum_{j=1}^{k}d_{\mathrm{ij}}^{-}}\;i=1,\ldots ,n,$$

with the example $z_{i}=\left(x_{i},y_{i}\right)$, where $d_{\mathrm{ij}}^{+}$ is the jth shortest distance from $x_{i}$ to other objects labelled the same as $x_{i}$, and $d_{\mathrm{ij}}^{-}$ is the jth shortest distance from $x_{i}$ to other objects labelled different from $x_{i}$. The parameter *k* is the number of elements taken into account. The larger $\alpha_{i}$ is, the stranger $z_{i}$ is, and it shows $z_{i}$ is more non-conformal than other elements.

### 2.3. Prediction in Online Mode

Online learning is the most popular and practical learning protocol in machine learning. In online learning mode, examples are presented one by one. Every time, we observe a new object $x_{i}$ and predict its label $y_{i}$ with old examples $\left(z_{1},\ldots ,z_{i-1}\right)$. Then, we observe the label $y_{i}$, and put $\left(x_{i},y_{i}\right)$ into the sequence of old examples. Then, we observe another new object $x_{i+1}$ and predict its label $y_{i+1}$ with old examples $\left(z_{1},\ldots ,z_{i}\right)$. The quality of prediction would be improved as more and more old examples are accumulated. This is the way of how the online mode are learning.

The process of conformal prediction can be summarized by the following protocol [10]:

$$\begin{array}{c} cONLINE\;PREDICTION\;PROTOCOL:\\ \;Err_{0}^{\epsilon }:=0,\epsilon \in \left(0,1\right);\\ \;Mult_{0}^{\epsilon }:=0,\epsilon \in \left(0,1\right);\\ \;Emp_{0}^{\epsilon }:=0,\epsilon \in \left(0,1\right);\\ \;Training\;set=\{\left(x_{1},y_{1}\right),\ldots ,\left(x_{i},y_{i}\right)\}\\ \;FOR\;n=l+1,l+2,\ldots :\\ \;Reality\;outputs\;x_{n}\in X;\\ \;Predictor\;outputs\;\Gamma_{n}^{\epsilon }\subseteq Y\;for\;all\;\epsilon \in \left(0,1\right)\\ \;Reality\;outputs\;y_{n}\in Y\\ \;err_{n}^{\epsilon }{=\{}_{0\;otherwise}^{1\;if\;y_{n}\notin \Gamma_{n}^{\epsilon }}\\ \;Err_{n}^{\epsilon }=Err_{n}^{\epsilon }+err_{n}^{\epsilon }\\ \;mult_{n}^{\epsilon }{=\{}_{0\;otherwise}^{1\;if|\Gamma_{n}^{\epsilon }|>1}\\ \;Mult_{n}^{\epsilon }=Mult_{n}^{\epsilon }+mult_{n}^{\epsilon }\\ \;emp_{n}^{\epsilon }{=\{}_{0\;otherwise}^{1\;if|\Gamma_{n}^{\epsilon }|=0}\\ \;Emp_{n}^{\epsilon }=Emp_{n}^{\epsilon }+emp_{n}^{\epsilon }\\ \;Training\;set=\{Training\;set,\left(x_{n},y_{n}\right)\}\\ \;End\;FOR. \end{array}$$

Combining Formula (5) and the strong law of large numbers, we can deduce that

(7) $$\lim_{n\to \infty }\sup\frac{{\mathrm{Err}}_{n}^{\epsilon }}{n}\leq \epsilon ,$$

holding with probability one for the conformal predictor [20].

### 2.4. Prediction in Offline Mode

Validity of conformal prediction is only proved for the online mode, in which every example is predicted one by one and every prediction is made based on the examples that have been considered before, rather than generating a certain rule from a fixed set of examples. However, the conformal prediction can still work in the offline mode and provide additional information about reliability for every prediction.

In offline mode, we can set a significance level to force the conformal predictor to output a prediction, which is the label with the highest *p*-value. This approach is called forcedprediction, similar to simpleprediction, which is designed to output singleton label. However, in this case, the significance level varies across different examples and the validity does not hold [19].

Two indicators, confidence and credibility, are used to provide additional information about the prediction. They are defined as:

$$\begin{array}{c} \mathrm{confidence}:\sup\{1-\epsilon :|\Gamma^{\epsilon }|\leq 1\}, \end{array}$$

$$\begin{array}{c} \mathrm{credibility}:\inf\{\epsilon :|\Gamma^{\epsilon }|=0\}. \end{array}$$

In the classification case, confidence equals 1 minus the second maximum *p*-value, which shows how confident we are rejecting other labels. Credibility equals the highest *p*-value, which shows how well the chosen label conforms to the rest of the set. The forced prediction is considered to be reliable if its confidence is close to 1 and credibility is not close to 0 [19].

## 3. Experiment and Methods

### 3.1. Sample Preparation

Nine categories of ginsengs (35 pieces for every category), which are showed in Table 1, were purchased from Changchun Medicinal Material Market (Changchun, China) randomly. Every ginseng sample was pulverized into powder, and 10 g powder of every sample was put into 100 mL empty glass bottles, which has been washed with clean air for 30 min separately before. Then, all of the bottles were sealed and placed at 50 °C for 30 min. Finally, 10 mL head-space gas was extracted from the top of the bottle for measurement with an injector.

### 3.2. E-Nose Equipment and Measurement

**Figure 1:**
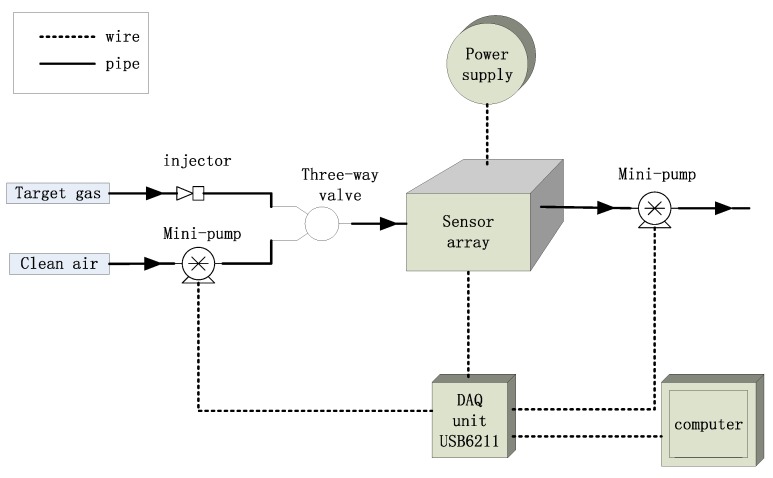
The schematics of the E-nose system.

All of the samples were measured with a homemade E-nose consisting of 16 metal-oxide semi-conductive sensors of TGS type purchased from Figaro Engineering Inc. (Osaka, Japan). TGS sensors have been used in the field of food classification [21,22]. The response characteristics of sensors are listed in Table 2. The schematic of E-nose system is shown in Figure 1. All sensors were fixed on a printed circuit board and placed in a 200 mL stainless chamber. A three-way valve was used to switch between target gas and clean dry air. Two mini vacuum pumps were fixed for air washing with a constant air flow of 1 L/min. A data acquisition (DAQ) unit USB6211, purchased from National Instruments Inc. (Austin, TX, USA), is equipped to record the signals of different sensors and control the pumps. Heating temperature for metal-oxide semi-conductive sensor is very important, and heater voltage of 5 V DC was applied for each sensor, as recommended by Figaro Engineering Inc. to guarantee the best performance of the sensors.

**Figure 2:**
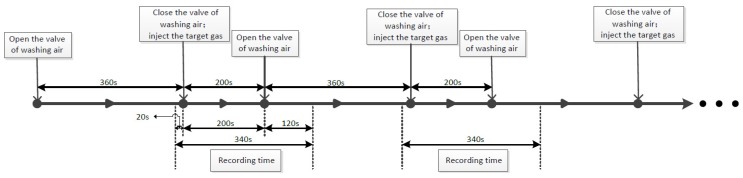
The procedure of measurement.

The procedure of measurement is as follows: firstly, the test chamber loaded with sensor array was washed by a clean-dry-air flow of 1 L/min for 360 s to allow the sensors to return to the baseline. Such flow can wash the sensors clean in 100–200 s, just near the time of reaction and won’t damage the E-nose. Then, washing-air flow was stopped and 10 mL target gas was taken and injected into the chamber through an injector. The target gas was left in the chamber obtained for 200 s, so that the gas sample diffused in the whole chamber freely to a steady condition. After that, the valve of washing-air flow was open to wash away the target gas. Responses of 16 sensors were recorded for 340 s, which included 20 s before the injection of the target gas, 200 s of the whole reaction time and 120 s after the washing flow was opened. The sampling frequency is of 10 Hz. The procedure of measurement is shown in Figure 2.

A total of 315 samples taken from nine categories of ginsengs were measured at room temperature (22–25 °C) and humidity (50–70%). During each measurement, the temperature and humidity of ambient environment were relatively stable.

Finally, all of the statistical analysis were performed by using Matlab 9.0.0.341360 (R2016a) (MathWorks, Natick, MA, USA).

### 3.3. Data Preprocessing

**Figure 3:**
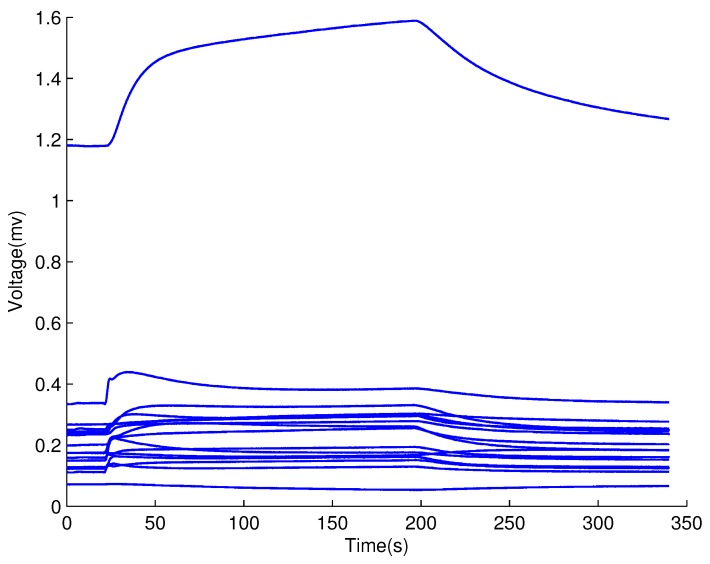
Typical response curves of 16 metal-oxide semi-conductive sensors to a sample.

The typical response curve of 16 sensors to a ginseng sample is shown in Figure 3. The voltage signal of each sensor was converted to resistance signal and then the resistance signal of each sensor was calibrated separately by:

(8) $$R=\left(R_{s}-R_{0}\right)/R_{0},$$

where $R_{s}$ is the original resistance signal and $R_{0}$ is the value of baseline. Five commonly used features were extracted (a total of 80 features with 16 sensors) and discussed as follows:

1. The maximal absolute response value, $R_{max}=max\left(|R|\right)$.
2. The area under the full response curve, $R_{int}=\int_{0}^{T}R\left(t\right)dt$, where T is the total measurement time, T = 340 s.
3–5. Exponential moving average of derivative of R, $E_{a}\left(R\right)=max\left(|y\left(k\right)|\right)$, k∈[1,400]. The discretely sampled exponential moving average is defined as y(k)=(1−a)y(k−1)+a(R(k))−R(k−1) with smoothing factors a=0.005,0.05,0.5 (sampling frequency: 10 Hz). Thus, three different smooth factors “*a*” give us the last three features.

## 4. Results and Discussion

### 4.1. Comparison of Forced Conformal Prediction with Simple Prediction

In offline mode, a forced conformal predictor can be used as a simple predictor to output only one label with the maximum *p*-value. It also provided indicators of confidence and credibility for the predicted label, compared to simple predictor. To compare the characteristics of forced conformal predictor and simple predictor, 1NN and 3NN were used as nonconformity measure methods and simple predictors separately.

Results of four typical individual predictions by forced predictor with CP-1NN are shown in Table 3. The *p*-value for each potential label, confidence, and credibility of forced conformal predictor are given. For Sample 1, both confidence and credibility are close to one, which means that we are confident of rejecting other potential labels, and the new object with predicted label conforms well to the old examples. This is the ideal case where we can strongly believe in our prediction. For Samples 2 and 3, the confidence is high (>0.90) while the credibility is rather low (<0.16). It tells us that the new object with a predicted label does not conform well to old examples. However, the new object with other potential labels conforms much worse to the old examples. Therefore, we have less confidence in rejecting other potential labels because no other label is appropriate or even close to the predicted label. For Sample 4, the confidence is low, which means that we are not so confident of rejecting other potential labels, so error prediction is very likely to occur in this case.

Compared to forced conformal prediction, simple prediction only outputs the predicted label with no comment on the reliability of the prediction.

In addition, we compare the average classification rate of forced conformal predictors and simple predictors. The average classification rate was achieved with leave-one-out validation: in every cycle, only one sample was taken as a testing set, and the remaining as a training set; the cycle was repeated until all samples had been treated as testing samples once. The result is shown in Table 4. We can see that when using forced prediction, the conformal predictors have the approximate classification accuracy as simple predictors, but provide additional information about the reliability of the prediction. Thus, we can demonstrate that the framework will not sacrifice the classification rate while it gives us prediction reliability information.

### 4.2. Validity of Online Conformal Prediction

**Figure 4:**
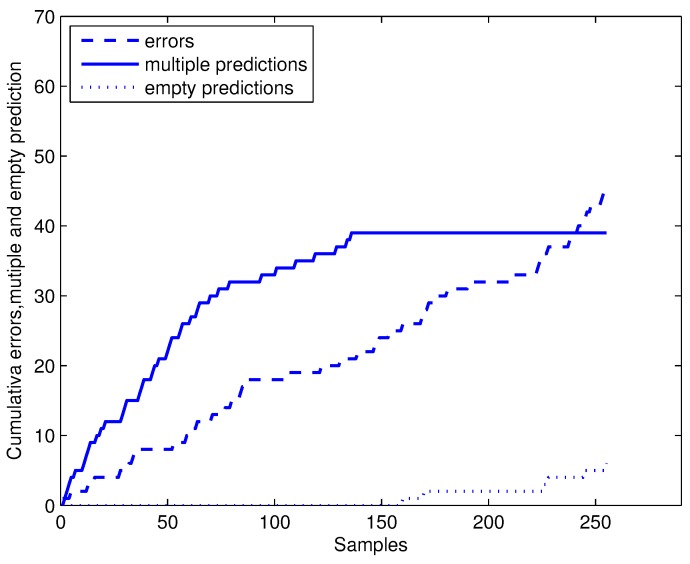
Online conformal prediction with confidence level of 80%.

CP-1NN and CP-3NN are used for online conformal prediction of discriminating ginseng samples according to the protocol in Section 2.3. Five samples were randomly taken from each class and treated as initial training sets. The rest of the samples were randomly sorted, predicted and added to the training set one by one. The cumulative error (${\mathrm{Err}}_{n}^{\epsilon }$) , multiple predictions (${\mathrm{Mult}}_{n}^{\epsilon }$) and empty predictions (${\mathrm{Emp}}_{n}^{\epsilon }$) for CP-1NN with confidence level of 80% are shown in Figure 4. As the number of samples in the training set increased, the predictor is more and more stable. Increasing of multiple predictions becomes slow gradually and even stops when the number of training samples is over 133. Then, the conformal predictor only outputs singleton prediction, just like a simple predictor dose, while keeping validity, which we will discuss below. The number of empty predictions starts to show up when the size of the training set is over 159, which can be explained by the fact that with enough training samples, the conformal predictor is confident of judging that these certain samples do not belong to any other class under the confidence level of 80%, and it is very likely that they are outliers.

**Figure 5:**
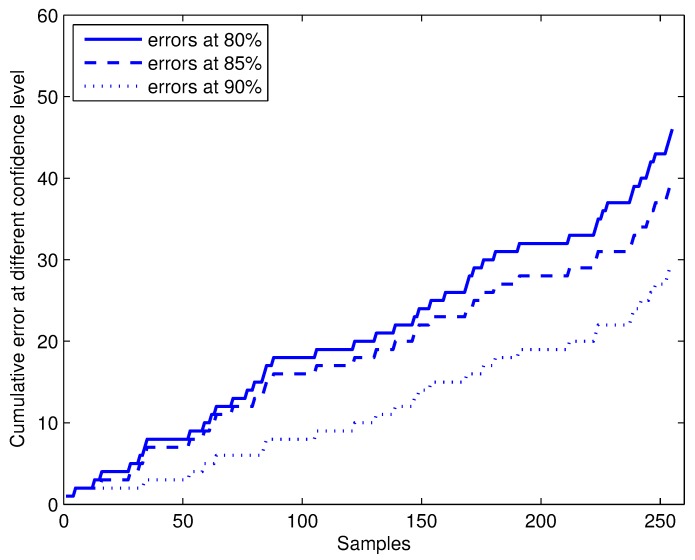
The cumulative errors of online prediction with CP-1NN (conformal prediction based on 1NN) at confidence levels of 80%, 85% and 90%.

Two main properties of conformal predictor are validity and efficiency. The ratio of errors ${\mathrm{Err}}_{n}^{\epsilon }/n$ is a measure of validity of conformal predictor. The cumulative error ${\mathrm{Err}}_{n}^{\epsilon }$ for CP-1NN with different confidence levels of 80%, 85% and 90% are shown in Figure 5. For all *n* and different confidence levels, we have ${\mathrm{Err}}_{n}^{\epsilon }<\epsilon n$ , which testify to the validity of online conformal predictors.

Besides guaranteeing the validity of conformal prediction, multiple prediction and empty prediction can provide the information about the distribution of the samples in feature space. For binary classification, multiple prediction with both labels may seem useless, but it demonstrates that both of the labels are reliable with a certain level of confidence, and this sample is very likely to be the borderline example between the two classes. For multi-label classification, multiple prediction can exclude those labels that are not reliable with a certain confidence level, and the sample is very likely to be the borderline example among the classes in this predicted region. Empty prediction tells that, with a certain confidence level, no label is reliable for the sample, and it is very likely to be an outlier.

### 4.3. Efficiency of Online Conformal Prediction

**Figure 6:**
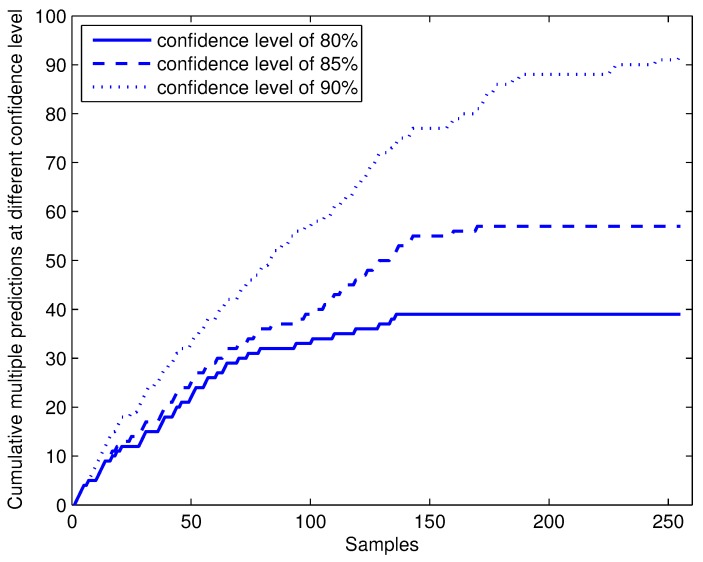
Cumulative multiple predictions of online conformal prediction with CP-1NN at different confidence levels of 80%, 85% and 90%.

By choosing different confidence levels 1−ϵ, the error ratio is guaranteed to be under significance level ϵ. However, it does not means that if we choose the higher confidence level, the predictor will perform better. As the predicted region by a conformal predictor is nested (stated in Section 2.1), higher confidence levels lead to low efficiency, which means wider predicted regions and more multiple predictions, and that is not what we expected. How the multiple predictions by online conformal predictors with CP-1NN vary with different confidence levels is showed in Figure 6. With lower confidence levels, conformal predictors can become stable with a smaller size of training set and gradually decreasing cases of multiple predictions. With higher confidence levels, the conformal predictor has to output more labels to avoid overrate errors occurring and more multiple prediction being output.

Thus, when the nonconformity measure method is fixed, we have to balance between confidence level and efficiency. However, we can still improve the efficiency of conformal predictors under the same confidence levels by improving the nonconformity measure methods. There are two major criteria depending on confidence level for conformal predictor [23]. One is the ratio of multiple predictions in the test sequence, which is known as M (which stands for ‘multiple’) criterion. The other is the average number of labels in the predict region of multiple prediction, which is known as the E (stand for ‘excess’) criterion. The lower these two criteria are, the better the efficiency of the predictor. These two criteria of efficiency for CP-1NN and CP-3NN with confidence levels of 80%, 85%, and 90% were compared in Table 5. The CP-1NN has the lowest ratio of multiple prediction for all confidence levels, and the average number of labels in multiple predictions is just a little higher than that of CP-3NN for confidence of 80% and 85%. Therefore, we can conclude that CP-1NN achieved better efficiency than that of CP-3NN.

## 5. Conclusions

In this work, conformal prediction is introduced for discriminating nine different ginsengs with a home-made electronic nose. Nonconformity measure methods with 1NN and 3NN are completed as underlying algorithms of conformal prediction. In offline mode, conformal predictors provides singleton predictions as well as additional information on reliability on the prediction. In addition, conformal prediction achieves a classification rate 84.44% with CP-1NN and 80.63% with CP-3NN, which are better than that of simple 1NN and 3NN with a modest increase, respectively. In online mode, the validity of conformal prediction is discussed and testified. The efficiency of conformal prediction are compared for CP-1NN and CP-3NN. Both the “M criterion” and “E criterion” of CP-1NN are lower than those of CP-3NN under every confidence level in the work, such as (15.29%, 1.23) of CP-1NN being lower than (23.92%, 1.32) at the confidence level of 80%, which indicates that the efficiency of CP-1NN is better than that of CP-3NN. The potential of conformal prediction for detecting borderline examples and outliers in the application of E-nose is also discussed. In conclusion, conformal prediction can give every prediction the estimation of reliability without sacrificing classification rates, which can make the prediction more comprehensive in both offline and online modes. Future work will be focused on designing new methods of computing nonconformity measures to improve the validity and efficiency of conformal prediction for E-nose data.

## Figures and Tables

**Table 1:** Details of the ginseng samples.

No.	Ginseng Samples	Places of Production
1	Chinese red ginseng	Ji’an
2	Chinese red ginseng	Fusong
3	Korean red ginseng	Ji’an
4	Chinese white ginseng	Ji’an
5	Chinese white ginseng	Fusong
6	American ginseng	Fusong
7	American ginseng	USA
8	American ginseng	Canada
9	American ginseng	Tonghua

**Table 2:** The response characteristics of sensors.

No.	Sensor Type	Response Characteristic
1	TGS800	Carbon monoxide, ethanol, methane, hydrogen, ammonia
2	TGS813	Carbon monoxide, ethanol, methane, hydrogen, isobutane
3	TGS813	Carbon monoxide, ethanol, methane, hydrogen, isobutane
4	TGS816	Carbon monoxide, ethanol, methane, hydrogen, isobutane
5	TGS821	Carbon monoxide, ethanol, methane, hydrogen
6	TGS822	Carbon monoxide, ethanol, methane, acetone, n-Hexane, benzene, isobutane
7	TGS822	Carbon monoxide, ethanol, methane, acetone, n-Hexane, benzene, isobutane
8	TGS826	Ammonia, trimethyl amine
9	TGS830	Ethanol, R-12, R-11, R-22, R-113
10	TGS832	R-134a, R-12 and R-22, ethanol
11	TGS800	Carbon monoxide, ethanol, methane, hydrogen, isobutane
12	TGS2620	Methane, Carbon monoxide, isobutane, hydrogen
13	TGS2600	Carbon monoxide, hydrogen
14	TGS2602	Hydrogen, ammonia ethanol, hydrogen sulfide, toluene
15	TGS2610	Ethanol, hydrogen, methane, isobutane/propane
16	TGS2611	Ethanol, hydrogen, isobutane, methane

**Table 3:** Typical individual prediction with CP-1NN (conformal prediction based on 1NN).

Sample Serial	True Lable	Forced Prediction	Confidence	Credibility	Simple Prediction
1	1	1	0.9968	0.9397	1
2	1	1	0.9047	0.1143	1
3	2	2	0.9587	0.1587	2
4	2	1	0.7714	0.2349	1

**Table 4:** Comparison of average classification rate of forced conformal predictors and simple predictors.

Predictors	1NN	3NN
Forced conformal predictor	84.44%	80.63%
Simple predictor	84.13%	77.46%

**Table 5:** Criterion of efficiency for online conformal predictors.

Confidence Level	CP-1NN		CP-3NN	
	M Criterion	E Criterion	M Criterion	E Criterion
80%	15.29%	1.23	23.92%	1.32
85%	22.35%	1.40	32.94%	1.47
90%	36.08%	1.62	45.88%	1.75
